# Anterior Versus Posterior Lumbar Interbody Fusion at L5-S1 in Hybrid Surgery for Adult Spinal Deformity: A Propensity Score Matching Analysis of Radiographic Results, Mechanical Complications, and Clinical Outcomes

**DOI:** 10.3390/jcm14051431

**Published:** 2025-02-20

**Authors:** Se-Jun Park, Dong-Ho Kang, Jin-Sung Park, Minwook Kang, Chong-Suh Lee, Kyunghun Jung

**Affiliations:** 1 Department of Orthopedic Surgery, Samsung Medical Center, Seoul 06351, Republic of Korea; sejunos@gmail.com (S.-J.P.); kang9451@gmail.com (D.-H.K.); paridot@hanmail.net (J.-S.P.); npng4eve@gmail.com (M.K.); 2 Department of Orthopedic Surgery, Haeundae Bumin Hospital, Busan 48094, Republic of Korea; csl3503@gmail.com

**Keywords:** adult spinal deformity, anterior lumbar interbody fusion, lumbosacral junction, L5-S1, posterior lumbar interbody fusion, propensity score matching analysis

## Abstract

**Objectives**: The aim of this study was to compare the radiographic results, mechanical complications, and clinical outcomes between anterior and posterior lumbar interbody fusion at L5–S1 (ALIF51 and PLIF51 groups, respectively) using a matched cohort of patients undergoing long fusion for adult spinal deformity (ASD). **Methods**: Patients who underwent hybrid surgery of ≥5-level fusion to the pelvis with a minimum follow-up duration of 2 years were included. The baseline characteristics of the groups were controlled using a propensity score matching analysis. The radiographic results, mechanical complications such as proximal junctional kyphosis/failure and metal failure, and clinical outcomes were compared between the groups. **Results**: In total, 79 patients were assigned to each group with comparable baseline data, except for a higher frequency of anterior column realignment procedures in the PLIF51 group than in the ALIF51 group (49.4% vs. 31.6%). At the last follow-up, L5–S1 segmental lordosis (SL) was significantly greater in the ALIF51 group than in the PLIF51 group (12.1° vs. 7.3°, *p* < 0.001). The final C7–sagittal vertical axis (SVA) was significantly smaller in the ALIF51 group than in the PLIF51 group (25.4 mm vs. 35.5 mm, *p* = 0.032). However, other global sagittal parameters were comparable between the groups. The mechanical complication rates, including metal failure at L5–S1, and the final clinical outcomes were comparable between the groups. **Conclusions**: ALIF51 has modest advantages over PLIF51 in terms of better restoring L5–S1 SL and C7–SVA with avoiding more invasive procedures above the L5–S1 levels. Other sagittal parameters, mechanical complication rates, and clinical outcomes did not differ between the groups.

## 1. Introduction

Adult spinal deformity (ASD) is a complex condition characterized by sagittal and/or coronal spinal malalignment, which leads to functional impairment, chronic low-back pain, negative perception of self-image, or psychological distress, all of which collectively impact patients’ quality of life [1]. In ASD surgery, hybrid surgery (i.e., multilevel lateral lumbar interbody fusion [LLIF] followed by posterior open surgery) can offer powerful corrective capacity for sagittal and coronal malalignment; thus, LLIF has been increasingly used in ASD surgery [1,2]. LLIF has several advantages over conventional osteotomy techniques in terms of less blood loss (from bone and epidural space) and a lower rate of neurologic complications without compromising the correction capacity [3,4,5]. Given that LLIF offers access only at the ≥ L4–5 level, the fusion method for the L5–S1 segment needs to be determined in cases where fusion extends to the sacrum or pelvis. Considering that the lumbosacral junction is mechanically weak, interbody arthrodesis (rather than posterior fusion) with pelvic fixation is strongly recommended for secure fusion achievement in case of long fusion surgery down to the sacrum for ASD [6,7,8].

Largely, there are two surgical options for lumbosacral interbody fusion: posterior lumbar interbody fusion (PLIF51) and anterior lumbar interbody fusion (ALIF51) according to the direction of cage insertion. ALIF51 has a distinct advantage over PLIF51 in terms of better restoration of segmental lordosis (SL) by releasing the anterior longitudinal ligament (ALL) and inserting the hyperlordotic cage into the disc space [9,10]. Unlike a single-level surgery, the advantages of ALIF51 over PLIF51 have not been clearly established in long fusion surgery for ASD. Theoretically, ALIF51 in ASD surgery also carries the same advantages as in a single-level surgery, but the clinical benefit of ALIF51 over PLIF51 has been reported inconsistently in terms of its radiographic results, mechanical failures, and clinical outcomes [6,7,11,12,13,14]. These controversial results may be attributed to the fact that the surgical outcomes after ASD surgery are not influenced solely by the specific fusion techniques at a single segment but complicatedly influenced by diverse patient- and surgery-related factors and global sagittal alignment. In addition, considering that highly heterogeneous properties usually exist with regard to baseline characteristics and surgical techniques in ASD surgery, the simple comparison of ALIF51 and PLIF51 without controlling these heterogeneities can bias the results.

Therefore, this study aims to compare the radiographic results, mechanical complications, and clinical outcomes between ALIF and PLIF at L5–S1 using a matched cohort of patients undergoing long fusion surgery for ASD. We hypothesized that the difference between ALIF and PLIF at L5-S1 would not be significant if the magnitude of total lumbar lordosis was achieved similarly following surgery.

## 2. Materials and Methods

### 2.1. Study Design and Ethical Considerations

This study was approved by our institutional review board. The requirement for informed consent was waived owing to the retrospective nature of this study.

### 2.2. Study Cohort

**Figure 1:**
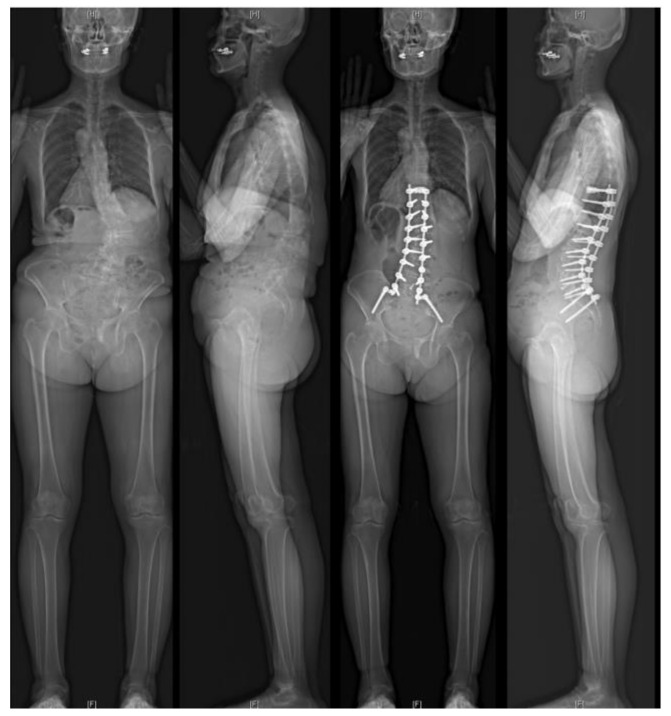
A representative case of the ALIF51 group. A 63-year-old female with severe kyphoscoliosis underwent deformity correction using multilevel LLIF at the ≥L4–5 levels and ALIF at L5–S1.

**Figure 2:**
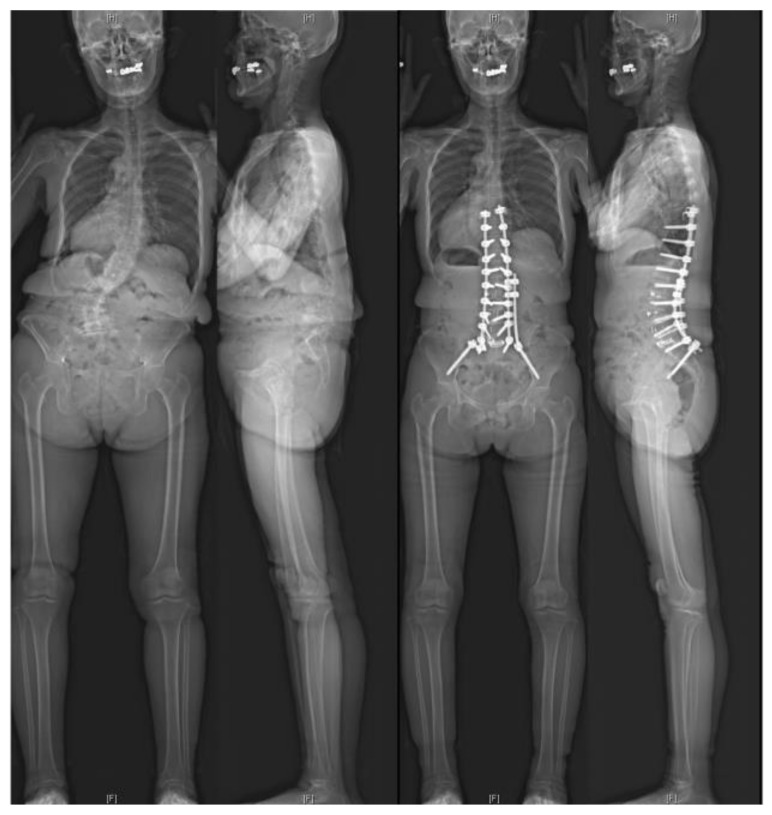
A representative case of the PLIF51 group. A 67-year-old female with severe kyphoscoliosis underwent corrective surgery using multilevel LLIF, including ACR, at the ≥L4–5 levels and PLIF at L5–S1.

The study cohort comprised consecutive patients who underwent long fusion surgery for ASD using the hybrid technique (i.e., LLIF followed by open posterior instrumentation) between 2014 and 2022. Patient data were retrieved from a prospective ASD database at our institution. The inclusion criteria are as follows: ASD radiographically defined as pelvic incidence (PI)–lumbar lordosis (LL) ≥ 10°, pelvic tilt (PT) ≥ 25°, C7–sagittal vertical axis (C7–SVA) ≥ 5 cm, or coronal Cobb angle ≥ 30°; ≥ 5-level fusion from the pelvis with ALIF or PLIF performed at L5–S1; and a minimum follow-up of 2 years. We excluded patients who underwent pedicle subtraction osteotomy (PSO) as the primary correcting maneuver for the following two reasons: (1) at our institute, hybrid surgery is a main surgical technique for ASD surgery, with only a small number of patients undergoing PSO, and (2) because PSO is reportedly associated with relatively high rates of postoperative complications [5], the inclusion of PSO may interfere with assessing the outcome measures according to L5–S1 fusion methods. In addition, patients with prior L5–S1 fusion, posterolateral fusion at L5–S1, and insufficient radiographic and clinical data were excluded. The patients were divided into the ALIF51 and PLIF51 groups according to the fusion methods at the L5–S1 levels. Representative cases are presented in Figure 1 and Figure 2.

### 2.3. Surgical Techniques

Three spine surgeons with ≥5-year clinical experience performed all surgeries. The choice of ALIF versus PLIF was made at the surgeon’s discretion considering vascular anatomy, previous abdominal surgery, and deformity status, even though ALIF is generally preferred over PLIF at our institution. ALIF was performed through midline and retroperitoneal approaches in the supine position during an earlier study period (before 2017). After that, ALIF was conducted in the lateral decubitus position during LLIF procedures. For this procedure, a separate incision was made 2–3 fingerbreadths anterior to the anterosuperior iliac spine. After the retroperitoneal space was assessed, retractors were placed to create the same working corridor (between the right and left iliac vessels) as in the supine ALIF procedure. After discectomy, a hyperlordotic cage filled with an allogenic chip bone graft and a demineralized bone matrix (DBM) was obliquely inserted from the ventral side. A buttress screw was inserted anterior to the cage to prevent anterior cage displacement. In the case of PLIF, one or two cages were used with a laminectomized chip bone graft and a DBM.

### 2.4. Baseline Data

Demographic data, including sex, age, body mass index, T-score (lowest score on spine or hip dual energy absorptiometry), and American Society of Anesthesiologists grade, and history of previous lumbar surgery were recorded. The surgical variables included number of LLIF levels, performance of anterior column realignment (ACR, modified LLIF technique with ALL release), total fusion levels, use of transverse process hook fixation at the uppermost instrumented vertebra +1, and postoperative follow-up duration. The preoperative radiographic data included PI, PI-LL, LL, sacral slope (SS), PT, thoracic kyphosis (TK), T1 pelvic angle (TPA), and C7–SVA. The total LL was evaluated separately for L1–5 lordosis and L5–S1 SL. To control the difference in baseline characteristics, a propensity score matching (PSM) analysis was conducted using the variables of sex, age, T-score, total fusion levels, and baseline PI-LL mismatch.

### 2.5. Outcome Measures

For the radiographic results, the same sagittal parameters as before surgery were evaluated at postoperative 6 weeks and at the last follow-up. In addition to the 6-week conventional sagittal parameters, the postoperative correction status was evaluated using global alignment assessment metrics including the global alignment proportion (GAP) and sagittal age-adjusted score (SAAS), which are known to be predictive for mechanical complications. The GAP score was classified according to the original scoring method [15] as follows: proportioned (≤2 points); moderately disproportioned (3–6 points); and severely disproportioned (≥7 points). The SAAS is a scoring system incorporating the three sagittal components of age-adjusted PI-LL, PT, and TPA [16]. After summing each score for the three components, the correction status was represented as an undercorrection (score ≤ −2), a matched correction (score between −1 and +1), and overcorrection (score ≥ +2). Regarding the mechanical complications, proximal junctional kyphosis/failure (PJK/F) and metal failures were evaluated. PJK refers to the proximal junctional angle (PJA) > 10° and ΔPJA > 10° according to previous definition [17]. PJF was defined as any case requiring revision surgery due to proximal junctional problems. Metal failures were evaluated for all segments within instrumentation in addition to the L5–S1 levels. Rod fractures, iliac screw-related complications, and revision surgery for metal failures were evaluated. Lastly, clinical outcomes were evaluated preoperatively and at the last follow-up using the following patient-reported outcome measures (PROM): the visual analog scale (VAS) for the back and leg, Oswestry Disability Index (ODI), and Scoliosis Research Society (SRS)-22 score. In addition, to compare the absolute values of the PROMs, we evaluated the minimal clinically important difference (MCID) for the aforementioned PROMs using the thresholds of 19.0 for back pain VAS, 15.0 for leg pain VAS, 18.9 for ODI, and 0.98 for SRS-22 subtotal score [18].

### 2.6. Statistical Analysis

Categorical variables are presented as frequency with percentages and were compared between the two groups using the chi-squared test. Continuous variables are presented as means with standard deviation and were compared between the two groups using the independent t-test or Wilcoxon rank-sum test. A professional statistician performed the statistical analyses using SPSS (version 27.0.0; IBM Corp., Armonk, NY, USA). Significance was set at *p*-value < 0.05.

## 3. Results

### 3.1. Baseline Data

A total of 200 patients were initially screened (Table 1). Before PSM, significant differences existed between the two groups with regard to sex, T-score, total fusion levels, PI-LL, LL, SS, PT, and TPA. Compared with the PLIF51 group, the ALIF51 group showed more female patients, lower T-score, longer fusion length, and more severe baseline deformity. After PSM using the variables of sex, age, T-score, total fusion levels, and preoperative PI-LL mismatch, 158 patients were included in the final analysis with 79 patients allocated in each group. All baseline demographic, surgical, and radiographic variables were comparable between the two groups, except for the rate of ACR procedures: ACR was performed more frequently in the PLIF51 group than in the ALIF51 group (49.4% vs. 31.6%, *p* = 0.023).

### 3.2. Radiographic Results

Regarding the 6-week radiographic results, most sagittal parameters were comparable between the two groups except for L1–5 lordosis, L5–S1 SL, and C7–SVA (Table 2). PI-LL and LL did not differ significantly between the two groups (3.6° vs. 4.8°, *p* = 0.422 for PI-LL; 48.5° vs. 47.9°, *p* = 0.716 for LL). However, L1–5 lordosis was significantly greater in the PLIF51 group than in the ALIF51 group (39.1° vs. 34.3°, *p* = 0.007), whereas L5–S1 SL was significantly greater in the ALIF51 group than in the PLIF51 group (14.3° vs. 8.8°, *p* < 0.001). C7–SVA was significantly less in the ALIF51 group than in the PLIF51 group (14.7 mm vs. 22.8 mm, *p* = 0.025). In addition to sagittal parameters, no significant differences were observed between the two groups with regard to patient distribution in the GAP score (*p* = 0.501) and SAAS systems (*p* = 0.475). At the last follow-up (mean, 42.0 months for the ALIF51 group and 36.9 months for PLIF51), similar results to the 6-week radiographic results were observed (Table 3). L1–5 lordosis was significantly greater in the PLIF51 group than in the ALIF51 group (35.9° vs. 31.8°, *p* = 0.038), while L5–S1 SL was significantly greater in the ALIF51 group than in the PLIF51 group (12.1° vs. 7.3°, *p* < 0.001). C7–SVA was significantly less in the ALIF51 group than in the PLIF51 group (25.4 mm vs. 35.5 mm, *p* = 0.032).

### 3.3. Mechanical Complications

Regarding PJK/F development, the rates of PJK and PJF were not significantly different between the ALIF51 and PLIF51 groups (50.6% vs. 41.8% for PJK, *p* = 0.273; 7.6% vs. 10.1% for PJK, *p* = 0.576) (Table 4). Regarding the metal failure, the rates of rod fracture did not significantly differ between the ALIF51 and PLIF51 groups either at the ≥L4–5 levels or at the L5–S1 levels (10.1% vs. 6.3% at the ≥L4–5 levels, 7.6% vs. 7.6% at the L5–S1 levels, *p* = 0.684) (Table 4). The rates of iliac screw-related complications, such as fracture and loosening, were also not significantly different between the two groups. Revision surgery for metal failure was performed in 10 patients (6.3%) of the entire cohort. The revision rates did not significantly differ between the two groups for all failure sites, including the L5–S1 levels.

### 3.4. Clinical Outcomes

The preoperative PROMs, including the VAS score for the back and leg, ODI, and total sum of the SRS-22 score, were not significantly different between the groups (Table 5). The clinical outcomes of all the PROMs at the last follow-up were also comparable between the ALIF51 and PLIF51 groups. Lastly, the proportion of patients who achieved MCID was comparable between the ALIF51 and PLIF51 groups (69.6% vs. 79.7% for back pain VAS, 51.9% vs. 54.4% for leg pain VAS, 58.2% vs. 50.6% for ODI, and 69.6% vs. 55.7% for SRS-22 subtotal).

## 4. Discussion

Given that the lumbosacral junction is mechanically weak as the base of a long construct in ASD surgery, the proper selection of a fusion method is crucial to minimize mechanical complications and potentiate the radiographic and clinical outcomes. ALIF has a distinct advantage over PLIF in terms of better restoration of segmental angle [9,10]. However, previous studies may introduce bias regarding the relative superiority of each procedure, likely due to a lack of control for baseline characteristics between the two groups. Thus, we performed a PSM analysis to control the baseline data between the two groups. In this study, we demonstrated that ALIF51 has modest advantages over PLIF51 in terms of better restoring L5–S1 SL and C7–SVA while avoiding more invasive procedures at the ≥L4–5 levels.

Previous studies have demonstrated the clear benefits of ALIF over PLIF in terms of radiographic and clinical aspects in an L5–S1 single-level surgery [9,10,19]. Lightsey et al. have demonstrated that ALIF showed superior restoration of SL compared with PLIF (change in SL, 11.3° vs. 1.3°), along with better clinical outcomes for patients with L5–S1 isthmic spondylolisthesis [10]. Kotani et al. have also reported that ALIF achieved a higher SL (17.0° vs. 11.5°) as well as superior fusion rates (97% vs. 92%) compared with PLIF [19]. However, in contrast to single-level surgery, the clinical benefit of ALIF over PLIF in long-segmental fusion for ASD has not been clearly established. In addition, the comparison of ALIF and PLIF at the L5–S1 levels in long fusion surgery may be more complex than in single-level surgery because diverse clinical factors, including baseline deformity status, surgical variables, and postoperative alignment correction status, can affect the surgical outcomes. For instance, when surgeons decide on the surgical techniques to be used at L5–S1, they may opt to choose ALIF for patients with more severe sagittal deformity, considering that ALIF is superior to PLIF in restoring the SL. In this context, in the current study, we observed that preoperative sagittal malalignment was more severe in the ALIF51 group than in the PLIF51 group before matching, showing greater PI-LL mismatch, smaller LL, smaller SS, greater PT, and greater TPA (Table 1). Considering that the baseline severity of sagittal malalignment affects the surgical outcomes differently [20], an uncontrolled baseline deformity status may give biased results when comparing ALIF and PLIF at the lumbosacral junction. In the same context, it has been reported that ALIF is superior in reducing the risks of pseudoarthrosis and subsequent metal failures [9,11]. However, lumbosacral pseudoarthrosis is affected not only by the fusion methods but also by other factors such as old age and longer fusion lengths [7,13,15]. Before PSM, we found that the total fusion levels were significantly greater in the ALIF51 group than in the PLIF51 group (8.2 vs. 7.0), with the pseudoarthrosis rates being likely to be higher in the ALIF51 group. These inhomogeneous properties in ASD patients and surgical techniques can lead to a bias, particularly when comparing a single surgical technique limited to only the L5–S1 segment. Therefore, we performed a PSM analysis to control different baseline characteristics among the groups using the variables that can affect the outcome measures for the current study, including sex, age, T-score, total fusion levels, and PI-LL mismatch. After PSM, all baseline variables were successfully controlled, except for the frequency of ACR.

With regard to the radiographic results, in agreement with a previous study, we also observed that L5–S1 SL was significantly greater in the ALIF51 group than in the PLIF51 group at 6 weeks (Table 2). Among the conventional radiographic parameters, a significant difference was found in C7–SVA, being less in the ALIF51 group than in the PLIF51 group (14.7 mm vs. 22.8 mm, *p* = 0.025), suggesting that ALIF is more advantageous in restoring global sagittal alignment compared to PLIF. Because the lever arm from the L5–S1 levels to C7 vertebra is the longest compared to from other segments, even small changes in L5–S1 SL can lead to substantial C7–SVA correction. These intergroup differences in L5–S1 SL and C7–SVA at postoperative 6 weeks were similarly observed at the last follow-up (Table 3).

It is noteworthy that the postoperative PI-LL or LL values, either at 6 weeks or at the last follow-up, were comparable between the ALIF51 and PLIF51 groups, despite the different L5–S1 SL values among the groups (Table 2 and Table 3). These results are because L1–5 lordosis was significantly greater in the PLIF51 group than in the ALIF51 group. In the same context, the ACR procedures were performed more frequently in the PLIF51 group than in the ALIF51 group (49.4% vs. 31.6%, *p* = 0.023 in Table 1). The ACR procedure is a modified technique used in conventional LLIF surgery, which is characterized by ALL release for the hyperlortodic cage [21,22]. Although ACR allows for greater correction of the segmental angle at the ≥L4–5 levels, ACR is now considered a more invasive technique due to the potential risks of vascular injury and mechanical complications, compared to conventional LLIF [22,23,24]. In the current study, ACR had to be performed more frequently in the PLIF51 group than in the ALIF51 group to compensate for the small SL gain at L5–S1 and to reach the same degree of correction in total LL as in the ALIF51 group. In this regard, ALIF51 has an advantage over PLIF51 in terms of avoiding more invasive procedures, such as ACR, at the levels above L5–S1 by creating greater SL at the L5–S1 segment.

In addition to the radiographic results, the issue of nonunion at the lumbosacral junction needs to be importantly addressed in ASD surgery because the L5–S1 levels are highly vulnerable to metal failure owing to the high biomechanical stresses inflicted on the most caudal end [6,7]. In the current study, the rates of metal failure and resultant revision surgery at the L5–S1 levels did not significantly differ between the ALIF51 and PLIF51 groups (Table 4). The pseudoarthrosis rates at the L5–S1 levels according to the fusion method (ALIF51 vs. PLIF51) in ASD surgery have been reported inconsistently. Buell et al. have reported that rod fractures at L5–S1 developed significantly more frequently in the PLIF51 group than in the ALIF51 group (28.6% and 7.1%) for patients with adult scoliosis [25]. Singh et al. have demonstrated a significantly higher risk of clinical pseudoarthrosis with PLIF than with ALIF in a univariate analysis (24.4% vs. 2.0%, *p* < 0.001), although this risk was not significant in the multivariate model [13]. However, Park et al. have reported no differences in the radiographic nonunion rates between the ALIF and PLIF groups based on 2-year computed tomography images [7]. The same authors have also demonstrated that the fusion method at L5–S1 (ALIF versus PLIF) did not affect the development of metal failure at the L5–S1 levels [6]. Furthermore, Meyer et al. showed that the ALIF group even had a trend toward a higher rate of nonunion and metal failure compared with the PLIF51 group in long-segment fusion for ASD [12]. One of the reasons for these contradictory results on lumbosacral nonunion issues may result from a lack of control of the inhomogeneous characteristics of the compared groups.

Finally, the clinical outcomes at the last follow-up were comparable between the two groups. These findings are attributed to comparable global sagittal parameters (e.g., PI-LL, PT, and TPA), which are known to affect the clinical outcomes [26,27,28], and comparable rates of mechanical complications. Although the final C7–SVA was significantly greater in the PLIF51 group than in the ALIF51 group (35.5 mm vs. 25.4 mm, *p* = 0.032), all of these values were within the optimal range (that is, <4 cm) according to the SRS-Schwab criteria [29]. Therefore, it seems that those differences in C7–SVA between the groups did not significantly differentiate the clinical outcomes between the groups.

### Limitation

This study has some limitations. First, this was a retrospective single-center study, which is an inherent limitation. Although all surgeries were performed under the widely accepted technical principles, there might be a difference in surgical skills for each procedure among the surgeons, potentially leading to expertise bias. In addition, this study was not a randomized controlled trial; thus, the selection bias in deciding ALIF versus PLIF is inevitably bound to exist. However, all baseline characteristics, including patient-specific, surgical, and radiographic variables, were successfully controlled in the groups after a propensity score matching process. Second, the study cohort predominantly comprised patients with UIV of the lower thoracic spine (mean total fusion level, 7.4 in the ALIF51 group and 7.0 in the PLIF51 group). This fusion level-related issue may limit the generalizability of our findings to patients receiving upper thoracic fusion surgery. Finally, ALIF51 was performed in different positions according to the study period (supine position before 2017 and lateral position thereafter). However, it is reported that SA restoration at the L5–S1 levels did not significantly differ between the supine and lateral ALIF procedures [30]. Future studies with a prospective, multicenter design using a larger study cohort are warranted to further elucidate the advantages of ALIF (vs. PLIF) at L5-S1.

## 5. Conclusions

ALIF51 has modest advantages over PLIF51 in terms of better restoring L5–S1 SL and C7–SVA while avoiding more invasive procedures at the ≥L4–5 levels. Although C7–SVA saw greater corrections in the ALIF51 group than in the PLIF51 group, the final C7–SVA values in both groups were within an optimal range. The rates of mechanical complications and the final clinical outcomes were comparable between the groups.

## Figures and Tables

**Table 1:** Baseline characteristics of overall cohorts.

Variables	Before Matching (N = 200)			After Matching (N = 158)		
	ALIF51 (N = 110)	PLIF51 (N = 90)	*p*	ALIF51 (N = 79)	PLIF51 (N = 79)	*p*
***Demographic data***						
Sex (female), n (%) *	98 (89.1%)	69 (76.7%)	**0.011**	64 (81.0%)	60 (75.9%)	0.439
Age (years) *	69.9 ± 6.8	69.9 ± 5.9	0.958	69.2 ± 7.6	69.8 ± 5.9	0.560
BMI (kg/m^2^)	25.9 ± 3.9	25.8 ± 3.9	0.421	25.6 ± 2.9	25.7 ± 3.9	0.799
T-score *	−1.5 ± 1.1	−1.2 ± 1.2	**0.048**	−1.4 ± 1.1	−1.2 ± 1.2	0.407
ASA grade	2.1 ± 0.4	2.1 ± 0.4	0.411	2.2 ± 0.4	2.1 ± 0.4	0.169
***Surgical data***						
Revision cases, n (%)	26 (23.6%)	16 (17.8%)	0.298	19 (24.1%)	14 (17.7%)	0.328
Number of LLIF levels, n (%)	2.6 ± 0.8	2.8 ± 0.6	0.196	2.6 ± 0.9	2.8 ± 0.6	0.193
ACR performance, n (%)	76 (49.4%)	39 (48.8%)	0.931	25 (31.6%)	39 (49.4%)	**0.023**
Total fusion levels *	8.2 ± 2.0	7.0 ± 2.1	**0.031**	7.4 ± 2.3	7.0 ± 2.1	0.127
TP hook at UIV + 1, n (%)	26 (23.6%)	13 (14.4%)	0.065	19 (24.1%)	11 (13.9%)	0.105
Follow-up duration (months)	44.1 ± 18.7	37.8 ± 16.0	0.097	42.0 ± 16.8	36.9 ± 12.3	0.145
***Radiographic data***						
PI (°)	53.4 ± 9.4	52.5 ± 11.3	0.528	52.1 ± 9.9	52.7 ± 11.3	0.744
PI-LL (°) *	40.5 ± 20.8	31.8 ± 18.0	**0.002**	33.5 ± 18.1	32.1 ± 17.9	0.624
LL (°)	12.9 ± 20.7	20.8 ± 17.6	**0.004**	18.7 ± 17.4	20.6 ± 17.6	0.483
L1–5 lordosis (°)	5.4 ± 20.5	12.5 ± 18.4	**0.010**	10.3 ± 17.5	12.4 ± 18.5	0.465
L5–S1 SA (°)	7.6 ± 7.9	8.3 ± 6.2	0.505	8.4 ± 6.5	8.2 ± 6.2	0.894
SS (°)	20.6 ± 11.2	23.7 ± 9.9	**0.038**	22.9 ± 10.4	23.6 ± 9.9	0.644
PT (°)	33.0 ± 11.6	28.7 ± 10.3	**0.006**	29.3 ± 10.6	29.0 ± 10.1	0.840
TK (°)	13.1 ± 15.5	15.9 ± 13.4	0.166	15.3 ± 13.1	15.9 ± 13.5	0.793
TPA (°)	31.9 ± 12.1	28.1 ± 12.4	**0.028**	27.9 ± 11.3	28.3 ± 12.4	0.836
C7–SVA (mm)	74.8 ± 55.6	65.9 ± 56.1	0.249	63.7 ± 52.5	65.4 ± 56.3	0.839

Bold *p* values indicate statistical significance. * Variables used for propensity score matching. ALIF51, anterior lumbar interbody fusion at L5–S1; PLIF51, posterior lumbar interbody fusion at L5–S1; BMI, body mass index, ASA; American Society of Anesthesiologists, LLIF, lateral lumbar interbody fusion; ACR, anterior column realignment; TP, transverse process; UIV, uppermost instrumented vertebra; PI, pelvic incidence; LL, lumbar lordosis; SA, segmental angle; SS, sacral slope; PT, pelvic tilt; TK, thoracic kyphosis; TPA, T1 pelvic angle; C7–SVA; C7–sagittal vertical axis.

**Table 2:** Comparison of 6-week postoperative radiographic results between the two groups.

Variables	ALIF51 (N = 79)	PLIF51 (N = 79)	*p*
***Sagittal parameters***			
PI (°)	53.7 ± 9.6	53.4 ± 10.8	0.859
PI-LL (°)	3.6 ± 9.9	4.8 ± 8.9	0.422
LL (°)	48.5 ± 10.2	47.9 ± 10.5	0.716
L1–5 lordosis (°)	34.3 ± 11.1	39.1 ± 10.8	**0.007**
L5–S1 SL (°)	14.3 ± 4.2	8.8 ± 3.6	**<0.001**
SS (°)	35.8 ± 8.2	34.6 ± 8.5	0.358
PT (°)	16.1 ± 8.0	17.9 ± 8.0	0.136
TK (°)	26.5 ± 11.2	28.8 ± 10.4	0.191
TPA (°)	13.4 ± 8.5	15.3 ± 7.3	0.452
C7–SVA (mm)	14.7 ± 22.1	22.8 ± 25.3	**0.025**
***Global alignment assessment metrics***			
GAP score			0.501
Proportioned, n (%)	32 (40.5%)	25 (31.6%)	
Moderately disproportioned, n (%)	34 (43.0%)	40 (50.6%)	
Severely disproportioned, n (%)	13 (16.5%)	14 (17.7%)	
SAAS			0.475
Undercorrection, n (%)	6 (7.6%)	5 (6.3%)	
Matched correction, n (%)	22 (27.8%)	16 (20.3%)	
Overcorrection, n (%)	51 (64.6%)	58 (73.4%)	

Bold *p* values indicate statistical significance. ALIF51, anterior lumbar interbody fusion at L5–S1; PLIF51, posterior lumbar interbody fusion at L5–S1; PI, pelvic incidence; LL, lumbar lordosis; SL, segmental lordosis; SS, sacral slope; PT, pelvic tilt; TK, thoracic kyphosis; TPA, T1 pelvic angle; C7–SVA; C7–sagittal vertical axis; GAP, global alignment proportion; SAAS, sagittal age-adjusted score.

**Table 3:** Comparison of radiographic results at the last follow-up between the two groups.

Sagittal Parameters	ALIF51 (N = 79)	PLIF51 (N = 79)	*p*
PI (°)	54.1 ± 9.6	52.8 ± 11.1	0.410
PI-LL (°)	10.2 ± 12.7	11.5 ± 11.7	0.480
LL (°)	43.9 ± 11.8	43.2 ± 12.5	0.160
L1–5 lordosis (°)	31.8 ± 13.1	35.9 ± 13.4	**0.038**
L5–S1 SL (°)	12.1 ± 5.0	7.3 ± 3.7	**<0.001**
SS (°)	32.5 ± 8.9	30.7 ± 8.8	0.204
PT (°)	21.7 ± 9.3	22.1 ± 8.4	0.758
TK (°)	30.4 ± 11.0	30.7 ± 12.3	0.863
TPA (°)	18.4 ± 8.4	22.9 ± 8.6	0.199
C7–SVA (mm)	25.4 ± 26.4	35.5 ± 31.8	**0.032**

Bold *p* values indicate statistical significance. ALIF51, anterior lumbar interbody fusion at L5–S1; PLIF51, posterior lumbar interbody fusion at L5–S1; PI, pelvic incidence; LL, lumbar lordosis; SL, segmental lordosis; SS, sacral slope; PT, pelvic tilt; TK, thoracic kyphosis; TPA, T1 pelvic angle; C7–SVA; C7–sagittal vertical axis.

**Table 4:** Comparison of mechanical complications between the two groups.

Mechanical Complications	ALIF51 (N = 79)	PLIF51 (N = 79)	*p*
***PJK/F***			
PJK			0.273
None, n (%)	39 (49.4%)	46 (58.2%)	
Yes, n (%)	40 (50.6%)	33 (41.8%)	
PJF, n (%)			0.576
None, n (%)	73 (92.4%)	71 (89.9%)	
Yes, n (%)	6 (7.6%)	8 (10.1%)	
***Metal failures***			
Rod fractures			0.684
None, n (%)	65 (82.3%)	68 (86.1%)	
At or above L4–5, n (%)	8 (10.1%)	5 (6.3%)	
At L5–S1, n (%)	6 (7.6%)	6 (7.6%)	
Iliac screw-related complications			0.873
None, n (%)	53 (67.1%)	50 (63.3%)	
Fracture, n (%)	3 (3.8%)	3 (3.8%)	
Loosening, n (%)	23 (29.1%)	26 (32.9%)	
Revision surgery for metal failures			0.444
None, n (%)	73 (92.4%)	75 (94.9%)	
For rod fracture at or above L4–5, n (%)	1 (1.3%)	2 (2.5%)	
For rod fracture at L5–S1, n (%)	4 (5.1%)	2 (2.5%)	
For iliac screw-related complication, n (%)	1 (1.3%)	0 (0%)	

Bold *p* values indicate statistical significance. ALIF51, anterior lumbar interbody fusion at L5–S1; PLIF51, posterior lumbar interbody fusion at L5–S1; PJK, proximal junctional kyphosis; PJF, proximal junctional failure.

**Table 5:** Comparison of clinical outcomes between the two groups.

PROM	ALIF51 (N = 79)	PLIF51 (N = 79)	*p*
***Preoperative***			
VAS for back pain	72.8 ± 17.2	71.2 ± 19.8	0.616
VAS for leg pain	64.2 ± 28.9	58.2 ± 30.3	0.232
ODI	57.2 ± 15.9	56.5 ± 16.1	0.787
SRS-22 subtotal	2.44 ± 0.55	2.41 ± 0.48	0.776
***At the last follow-up***			
VAS for back pain	38.8 ± 26.2	31.1 ± 25.0	0.093
VAS for leg pain	43.1 ± 30.3	36.6 ± 30.8	0.172
ODI	36.3 ± 19.8	30.9 ± 19.7	0.128
SRS-22 subtotal	3.66 ± 0.81	3.79 ± 0.81	0.324
***Proportion of patients achieving MCID***			
VAS for back pain, n (%)	55 (69.6%)	63 (79.7%)	0.113
VAS for leg pain, n (%)	41 (51.9%)	43 (54.4%)	0.731
ODI, n (%)	46 (58.2%)	40 (50.6%)	0.371
SRS-22 subtotal, n (%)	55 (69.6%)	44 (55.7%)	0.140

Bold *p* values indicate statistical significance. PROM, patient-reported outcome measure; ALIF51, anterior lumbar interbody fusion at L5–S1; PLIF51, posterior lumbar interbody fusion at L5–S1; VAS, visual analogue scale; ODI, Oswestry Disability Index; SRS, Scoliosis Research Society.

## Data Availability

The data underlying this article cannot be shared publicly because of the privacy of the individuals who participated in this study. The data can be shared by the corresponding authors upon reasonable request.

## References

[1] Kim H.J., Yang J.H., Chang D.G., Lenke L.G., Suh S.W., Nam Y., Park S.C., Suk S.I. (2022). Adult Spinal Deformity: A Comprehensive Review of Current Advances and Future Directions. Asian Spine J..

[2] Fujibayashi S., Takemoto M., Ishii K., Funao H., Isogai N., Otsuki B., Shimizu T., Nakamura T., Matsuda S. (2022). Multicenter Prospective Study of Lateral Lumbar Interbody Fusions Using Bioactive Porous Titanium Spacers without Bone Grafts. Asian Spine J..

[3] Iwamae M., Matsumura A., Namikawa T., Kato M., Hori Y., Yabu A., Sawada Y., Hidaka N., Nakamura H. (2020). Surgical Outcomes of Multilevel Posterior Lumbar Interbody Fusion versus Lateral Lumbar Interbody Fusion for the Correction of Adult Spinal Deformity: A Comparative Clinical Study. Asian Spine J..

[4] Yang H., Liu J., Hai Y., Han B. (2023). What Are the Benefits of Lateral Lumbar Interbody Fusion on the Treatment of Adult Spinal Deformity: A Systematic Review and Meta-Analysis Deformity. Glob. Spine J..

[5] Chi J., Zhang Y., Fontaine A., Zhang Z., Wang J., Labaran L., Li X. (2024). Pedicle Subtraction Osteotomy Versus Multilevel Anterior Lumbar Interbody Fusion and Lateral Lumbar Interbody Fusion in the Treatment of Adult Spinal Deformity: Trends, Outcomes, and Cost. Clin. Spine Surg..

[6] Park S.J., Park J.S., Nam Y., Yum T.H., Choi Y.T., Lee C.S. (2021). Failure Types and Related Factors of Spinopelvic Fixation After Long Construct Fusion for Adult Spinal Deformity. Neurosurgery.

[7] Park S.J., Lee C.S., Park J.S., Yum T.H., Shin T.S., Chang J.W., Lee K.H. (2022). L5-S1 nonunion occurrence even after anterior column support combined with iliac screw fixation in long fusion for adult spinal deformity: CT-based analysis at 2-year follow-up. J. Neurosurg. Spine.

[8] Lee C.S., Chung S.S., Choi S.W., Yu J.W., Sohn M.S. (2010). Critical length of fusion requiring additional fixation to prevent nonunion of the lumbosacral junction. Spine.

[9] Kotani Y., Ikeura A., Tokunaga H., Saito T. (2021). Single-level controlled comparison of OLIF51 and percutaneous screw in lateral position versus MIS-TLIF for lumbosacral degenerative disorders: Clinical and radiologic study. J. Orthop. Sci..

[10] Lightsey H.M.T., Pisano A.J., Striano B.M., Crawford A.M., Xiong G.X., Hershman S., Schoenfeld A.J., Simpson A.K. (2022). ALIF Versus TLIF for L5-S1 Isthmic Spondylolisthesis: ALIF Demonstrates Superior Segmental and Regional Radiographic Outcomes and Clinical Improvements Across More Patient-reported Outcome Measures Domains. Spine.

[11] Kotani Y., Ikeura A., Tanaka T., Saito T. (2024). Clinical and Radiologic Analysis of Minimally Invasive Anterior-Posterior Combined Surgery for Adult Spinal Deformity: Comparison of Oblique Lateral Interbody Fusion at L5/S1 (OLIF51) versus Transforaminal Interbody Fusion. Medicina.

[12] Meyers A.J., Wick J.B., Rodnoi P., Khan A., Klineberg E.O. (2021). Does L5-S1 Anterior Lumbar Interbody Fusion Improve Sagittal Alignment or Fusion Rates in Long Segment Fusion for Adult Spinal Deformity?. Glob. Spine J..

[13] Singh V., Oppermann M., Evaniew N., Soroceanu A., Nicholls F., Jacobs W.B., Thomas K., Swamy G. (2023). L5-S1 Pseudoarthrosis Rate with ALIF Versus TLIF in Adult Spinal Deformity Surgeries: A Retrospective Analysis of 100 Patients. World Neurosurg..

[14] Adogwa O., Buchowski J.M., Lenke L.G., Shlykov M.A., El Dafrawy M., Lertudomphonwanit T., Obey M.R., Koscso J., Gupta M.C., Bridwell K.H. (2020). Comparison of rod fracture rates in long spinal deformity constructs after transforaminal versus anterior lumbar interbody fusions: A single-institution analysis. J. Neurosurg. Spine.

[15] Yilgor C., Sogunmez N., Boissiere L., Yavuz Y., Obeid I., Kleinstuck F., Perez-Grueso F.J.S., Acaroglu E., Haddad S., Mannion A.F. (2017). Global Alignment and Proportion (GAP) Score: Development and Validation of a New Method of Analyzing Spinopelvic Alignment to Predict Mechanical Complications After Adult Spinal Deformity Surgery. J. Bone Jt. Surg. Am..

[16] Lafage R., Smith J.S., Elysee J., Passias P., Bess S., Klineberg E., Kim H.J., Shaffrey C., Burton D., Hostin R. (2022). Sagittal age-adjusted score (SAAS) for adult spinal deformity (ASD) more effectively predicts surgical outcomes and proximal junctional kyphosis than previous classifications. Spine Deform..

[17] Glattes R.C., Bridwell K.H., Lenke L.G., Kim Y.J., Rinella A., Edwards C. (2005). Proximal junctional kyphosis in adult spinal deformity following long instrumented posterior spinal fusion: Incidence, outcomes, and risk factor analysis. Spine.

[18] Yuan L., Li W., Zeng Y., Chen Z. (2023). Minimum Clinically Important Difference in Patient-reported Outcome Measures in de novo Degenerative Lumbar Scoliosis: Is it Appropriate to Apply the Values of Adult Spine Deformity?. Spine.

[19] Mun H.Y., Ko M.J., Kim Y.B., Park S.W. (2020). Usefulness of Oblique Lateral Interbody Fusion at L5-S1 Level Compared to Transforaminal Lumbar Interbody Fusion. J. Korean Neurosurg. Soc..

[20] Kwon O., Lee S., Park S.M., Yeom J.S., Kim H.J. (2022). A Complement Type to SRS-Schwab Adult Spinal Deformity Classification: The Failure of Pelvic Compensation. Spine.

[21] Uribe J.S., Smith D.A., Dakwar E., Baaj A.A., Mundis G.M., Turner A.W., Cornwall G.B., Akbarnia B.A. (2012). Lordosis restoration after anterior longitudinal ligament release and placement of lateral hyperlordotic interbody cages during the minimally invasive lateral transpsoas approach: A radiographic study in cadavers. J. Neurosurg. Spine.

[22] Deukmedjian A.R., Le T.V., Baaj A.A., Dakwar E., Smith D.A., Uribe J.S. (2012). Anterior longitudinal ligament release using the minimally invasive lateral retroperitoneal transpsoas approach: A cadaveric feasibility study and report of 4 clinical cases. J. Neurosurg. Spine.

[23] Cheung Z.B., Chen D.H., White S.J.W., Kim J.S., Cho S.K. (2019). Anterior Column Realignment in Adult Spinal Deformity: A Case Report and Review of the Literature. World Neurosurg..

[24] Park S.J., Park J.S., Kang M., Jung K., Lee C.S., Kang D.H. (2025). Incidence and Risk Factors for Mechanical Failure After Anterior Column Realignment in Adult Spinal Deformity Surgery. Spine.

[25] Buell T.J., Shaffrey C.I., Bess S., Kim H.J., Klineberg E.O., Lafage V., Lafage R., Protopsaltis T.S., Passias P.G., Mundis G.M. (2021). Multicenter assessment of outcomes and complications associated with transforaminal versus anterior lumbar interbody fusion for fractional curve correction. J. Neurosurg. Spine.

[26] Schwab F., Ungar B., Blondel B., Buchowski J., Coe J., Deinlein D., DeWald C., Mehdian H., Shaffrey C., Tribus C. (2012). Scoliosis Research Society-Schwab adult spinal deformity classification: A validation study. Spine.

[27] Lafage V., Schwab F., Patel A., Hawkinson N., Farcy J.P. (2009). Pelvic tilt and truncal inclination: Two key radiographic parameters in the setting of adults with spinal deformity. Spine.

[28] Protopsaltis T., Schwab F., Bronsard N., Smith J.S., Klineberg E., Mundis G., Ryan D.J., Hostin R., Hart R., Burton D. (2014). The T1 Pelvic Angle, a Novel Radiographic Measure of Global Sagittal Deformity, Accounts for Both Spinal Inclination and Pelvic Tilt and Correlates with Health-Related Quality of Life. J. Bone Jt. Surg..

[29] Slattery C., Verma K. (2018). Classification in Brief: SRS-Schwab Classification of Adult Spinal Deformity. Clin. Orthop. Relat. Res..

[30] Xi Z., Burch S., Mummaneni P.V., Chang C.C., Ruan H., Eichler C., Chou D. (2020). Supine anterior lumbar interbody fusion versus lateral position oblique lumbar interbody fusion at L5-S1: A comparison of two approaches to the lumbosacral junction. J. Clin. Neurosci..

